# Biplane interventional pediatric system with cone‐beam CT: dose and image quality characterization for the default protocols

**DOI:** 10.1120/jacmp.v17i4.5828

**Published:** 2016-07-08

**Authors:** Eva Corredoira, Eliseo Vañó, Luis Alejo, Carlos Ubeda, Federico Gutiérrez‐Larraya, Julia Garayoa

**Affiliations:** ^1^ Medical Physics and Radiation Protection Service, Hospital Universitario La Paz Madrid Spain; ^2^ Radiology Department, Faculty of Medicine Universidad Complutense and IdIS, Hospital Universitario San Carlos Madrid Spain; ^3^ Medical Technology Department Radiological Sciences Center, Health Sciences Faculty, Tarapacá University Arica Chile; ^4^ Pediatric Cardiology Department Hospital Universitario La Paz Madrid Spain; ^5^ Radiation Protection Service, Hospital Universitario Fundación Jiménez Díaz Madrid Spain

**Keywords:** cone‐beam CT, dosimetry, image quality, pediatric cardiac catheterization, rotational angiography

## Abstract

The aim of this study was to assess image quality and radiation dose of a biplane angiographic system with cone‐beam CT (CBCT) capability tuned for pediatric cardiac procedures. The results of this study can be used to explore dose reduction techniques. For pulsed fluoroscopy and cine modes, polymethyl methacrylate phantoms of various thicknesses and a Leeds TOR 18‐FG test object were employed. Various fields of view (FOV) were selected. For CBCT, the study employed head and body dose phantoms, Catphan 504, and an anthropomorphic cardiology phantom. The study also compared two 3D rotational angiography protocols. The entrance surface air kerma per frame increases by a factor of 3–12 when comparing cine and fluoroscopy frames. The biggest difference in the signal‐to‐noise ratio between fluoroscopy and cine modes occurs at FOV 32 cm because fluoroscopy is acquired at a 1440×1440 pixel matrix size and in unbinned mode, whereas cine is acquired at 720×720 pixels and in binned mode. The high‐contrast spatial resolution of cine is better than that of fluoroscopy, except for FOV 32 cm, because fluoroscopy mode with 32 cm FOV is unbinned. Acquiring CBCT series with a 16 cm head phantom using the standard dose protocol results in a threefold dose increase compared with the low‐dose protocol. Although the amount of noise present in the images acquired with the low‐dose protocol is much higher than that obtained with the standard mode, the images present better spatial resolution. A 1 mm diameter rod with 250 Hounsfield units can be distinguished in reconstructed images with an 8 mm slice width. Pediatric‐specific protocols provide lower doses while maintaining sufficient image quality. The system offers a novel 3D imaging mode. The acquisition of CBCT images results in increased doses administered to the patients, but also provides further diagnostic information contained in the volumetric images. The assessed CBCT protocols provide images that are noisy, but with very good spatial resolution.

PACS number(s): 87.59.‐e, 87.59.‐C, 87.59.‐cf, 87.59.Dj, 87.57. uq

## I. INTRODUCTION

The International Commission on Radiological Protection and the European Commission^(1–3)^require interventional X‐ray systems to undergo a series of tests prior to use to ensure that the equipment performs satisfactorily in clinical practice. These tests cover two main aspects: 1) the entrance surface air kerma (ESAK) of an appropriate phantom under normal operating conditions, simulating various patient thicknesses in the commonly used imaging modes: fluoroscopy, cine and, more recently, the three‐dimensional rotational angiography (3D‐RA), also known as cone‐beam CT (CBCT); and 2) image quality assessment (using test objects) for the various imaging protocols used in clinical practice. This physical characterization helps cardiologists select the best protocols and operation modes with sufficient image quality (and appropriate dose) to guide and document the procedures.^4^, ^5^ This part of the commissioning testing sets the baseline values as a reference for future routine quality control tests (constancy tests) to monitor the most significant system operation parameters to ensure their stability over time.^(2,3,6)^A recent study that included 756 pediatric cardiac catheterization procedures concluded that the percentage increases in the median value of the air kerma‐area product due to CBCT were 33% and 16% for diagnostic and therapeutic procedures, respectively^(7)^ To improve optimization and properly manage patient doses with sufficient diagnostic‐quality imaging, knowledge of the equipment's performance is necessary. This report presents the results of the physical characterization conducted as part of the commissioning of a biplane angiography system dedicated to pediatric interventional cardiology (IC), including the 3D‐RA mode as one of the imaging options. These tests were performed in addition to the patient dose survey because equipment performance and setup are factors contributing to patient dose variability. The results can be used to explore optimization strategies to reduce the dose to a level such that the dose does not compromise the image quality required for the best clinical outcome.

## II. MATERIALS AND METHODS

The study employed a Siemens Artis Zee VC14 biplane angiographic X‐ray system (Siemens AG, Munich, Germany), equipped with two 100 kW generators at 125 kV, which was customized for pediatric IC procedures. The system was equipped with two flat amorphous silicon detectors with cesium iodide scintillators consisting of a frontal detector measuring 30×38 cm (48 cm diagonal), with a pixel size of 154 μm, and a 20×20 cm (25 cm diagonal) lateral detector, with a pixel size of 184 μm. This system was one of the first installed in Europe and it enables the acquisition of CBCT images for pediatric IC. This unit offers a combination of real‐time fluoroscopic and near real‐time tomographic imaging of heart anatomy. Interventional cardiologists can combine high‐resolution, cross‐sectional CBCT morphological data with the convenience and speed of standard angiography.

### A. Cine and fluoroscopy modes

Two fluoroscopy protocols (Ped <12 kg and FL3040) and two cine acquisition modes (Card <12 kg and LV3040 [left ventricle]) were selected for pediatric cardiac applications. Those modes and protocols were studied using the methodology agreed upon during the SENTINEL and DIMOND European programs.^8^, ^9^

The default fluoroscopy mode is 10 pulses per second in both protocols; however, cardiologists who are trained and certified in radiological protection according to national regulations routinely use fluoroscopy modes with 3 pulses per second to reduce patient dose. In general, cardiac studies on children require higher pulse rates than those in adults because of the faster heart rate of children, but cardiologists can reduce the frame rate when image quality is not a concern — for example, during fluoroscopy runs when introducing and manipulating catheters. In cine mode, the default configuration is 30 frames per second, which is used routinely. The equipment was adjusted for the “Ped<12 kg” fluoroscopy protocol with an incident air kerma^(10)^at the entrance of the image detector set to 0.036 μGy per frame and to 0.045 μGy per frame for the FL3040 protocol. Pediatric cine acquisition was configured with an incident air kerma at the entrance of the image detector set to 0.109 μGy per frame, and the left ventricle protocol acquisition was set to 0.17 μGy per frame. Additional filtration (automatic in this system) from a 0.1−0.9 mm copper filter and virtual collimation were available. The X‐ray system uses a dynamic copper filtration system to reduce low‐energy radiation in the X‐ray beam. The automatic exposure control adjusts the copper filtration without any user interaction. For every fluoroscopic and acquisition protocol, there are settings that determine the desired tube potential plateau and the minimum and maximum copper filter thickness. Thus, in addition to the copper filtration, the system also adapts the tube potential to the patient thickness to maintain a constant image quality. When the protocol is selected, the copper filter is moved into position before the first images are taken. During the procedure, the prefilter setting is based on a real‐time absorption measurement of the object in the beam. Whenever the tube potential required for penetrating the patient exceeds the predefined tube potential threshold, copper is removed until the given limits are met. This tube potential threshold helps maintain a good image contrast.^(11)^ The distance from the isocenter to the floor was 107 cm, and the distance from the focus to the isocenter was 75 cm.

### B. Cone‐beam CT mode

The CBCT acquisition was performed using the frontal detector, acquiring the image of the whole volume of interest in a single (partial) rotation of the source and detector. The volumetric image acquisition was performed using the following parameters: 200° rotation, with an angulation step of 1.5°; projection on a 30×40 cm flat‐panel detector, with a field of view (FOV) of 48 cm or 42 cm (diagonal dimension) and an X‐ray source detector distance of 120 cm. Two 3D cardiac examination modes were set for the system: 5sDRc (standard‐dose protocol) and 5sDR‐L (low‐dose protocol). For both protocols, the default examination settings were 26.6 frames/s and a 5 s acquisition time. The tube current, kVp, and pulse width are determined by the equipment using fluoroscopy images obtained just before the 3D‐RA run. The standard‐dose protocol employs the large focal size (1 mm), no additional copper filter, and an incident air kerma at the entrance of the image detector set at 0.36 μGy per image. The low‐dose protocol uses the small focal size (0.6 mm), a 0.1 mm copper filter, and an incident air kerma at the entrance of the detector set at 0.10 μGy per image. Those are the exam set by the installation engineers.

### C. Dosimetry and image quality for cine and fluoroscopy modes

**Figure 1:**
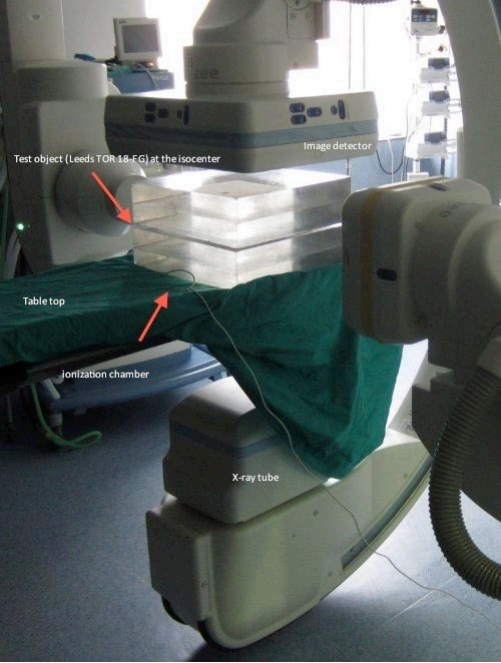
Experimental arrangements to measure incident air kerma and image quality using a PMMA slab phantom. A flat ionization chamber in contact with the PMMA plates was employed to measure incident air kerma. To evaluate the image quality during dose measurements, the test object was always positioned at mid‐PMMA thickness at the isocenter (the table was lowered when the PMMA thickness was increased).

Various chest thicknesses for patients were simulated using 4 to 20 cm polymethyl methacrylate (PMMA) plates measuring 25×25×5 cm (or 1 or 2 cm thickness). According to Rassow et al.,^(12)^ the ratio of patient chest thickness to PMMA thickness is approximately 1.5. Thus chest thicknesses from 6 to 30 cm in steps of 6 cm were simulated. To evaluate the image quality during fluoroscopy and cine acquisitions, a Leeds test object (TO) TOR 18FG^(13)^ was employed. The TO contains 18 low‐contrast circles each with varying metal thicknesses and exponential changes in contrast from one circle to the next. The TO also includes a standard metal bar pattern in the center to measure high‐contrast spatial resolution (HCSR), with 21 sets of bars whose resolution ranges from 0.5 to 5 lp/mm. To evaluate the image quality during dose measurements (thereby simulating clinical conditions), the TO was always positioned at mid‐PMMA thickness at the isocenter (the table was lowered when the PMMA thickness was increased). The image detector was always positioned approximately 8 cm from the last PMMA plate, which is similar to clinical practice with patients (Fig. 1). For an FOV of 22 cm, only 4 and 8 cm of PMMA were employed to compare fluoroscopy and cine acquisition modes. Due to the physical size of the flat ionization chamber used (9.17 cm diameter), smaller fields of view could not be measured. For the FOV of 48 cm, the pediatric protocols for patients weighing less than 12 kg were not evaluated because in pediatric cardiac studies, smaller FOVs are selected due to the small size of the volume of interest.

A flat ionization chamber (model 10x5–60) with a Radcal 9015 radiation meter (Radcal Corp., Monrovia, CA) in contact with the PMMA plates was employed to measure ESAK.^(10)^ The flat ionization chamber was duly calibrated by official calibration laboratories. The ionization chamber has an energy dependence of less than 5% for the employed energy range. The images were recorded simultaneously with the dose measurements.

To evaluate the changes in image quality for the various PMMA thicknesses and acquisition modes, all image series for cine and fluoroscopy were recorded in DICOM format at 1024×1024 pixels and 12 bits.

**Figure 2:**
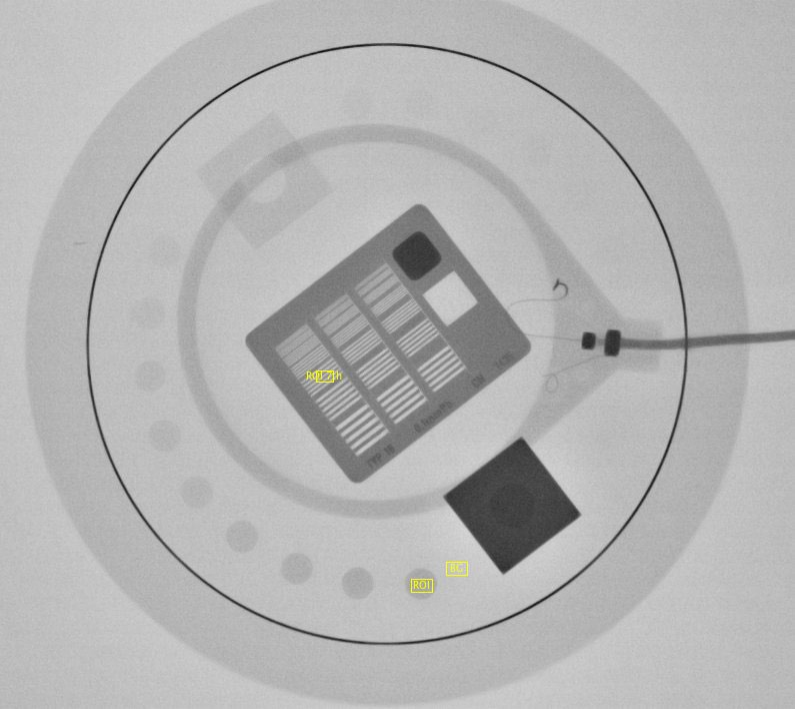
Image corresponds to LV3040 acquisition mode 48 cm FOV and 8 cm PMMA. The image shows the region of interest (ROI), background (BG), and ROI inside the seventh group in the high‐contrast spatial resolution area (${\text{ROI}}_{7\text{th}}$) employed to perform the objective image quality evaluation.

The image quality assessment was performed using ImageJ^(14)^ software (version 1.48r)^(15)^by averaging three images in each set (specifically, images 5, 8 and 10 in the series). To ensure the measurement stability of the tube output, we excluded the first four images. We always selected the same region of interest (ROI) to measure mean pixel values and standard deviation (SD). The evaluation was performed specifically in the first rod with the highest contrast (Fig. 2) and in an adjacent region outside the rods (background [BG]). The image comparison was relative, and the choice of rod was irrelevant; however, we selected the first one to reduce the measurement deviations. The spatial resolution was evaluated with an ROI in the seventh group of the bars pattern in the HCSR area. As before, the selection of the seventh group was irrelevant; we chose an intermediate group for comparison purposes.

The image quality was evaluated using the following numerical parameters: signal‐to‐noise ratio (SNR) for low‐contrast evaluation; 1 figure of merit (FOM), which indicates the necessary dose to obtain a certain image quality, using the SNR2 parameter; and high‐contrast spatial resolution (HCSR).^16^, ^17^ These parameters are defined as:

(1) $$SNR=\frac{BG-ROI}{\sqrt{\frac{SD_{ROI}^{2}+SD_{BG}^{2}}{2}}}$$

(2) $$FOM=\frac{SNR^{2}}{ESAK}$$

where, *BG* is the mean pixel value in an ROI at the background area, *ROI* is the mean pixel value in an ROI in the first rod with highest contrast, $SD_{ROI}$ is the standard deviation of the pixel values in an ROI in the first circle with highest contrast, and $SD_{BG}$ is the standard deviation of the pixel values in an ROI in the background area. As a representative of HCSR we choose the standard deviation of the pixel values in an ROI inside the seventh group (${\text{SD}}_{7\text{th}}$) in the HCSR pattern (${\text{SD}}_{\text{ROI}7}{}^{\text{th}}$).

### D. Dosimetry for the cone‐beam CT mode

**Figure 3:**
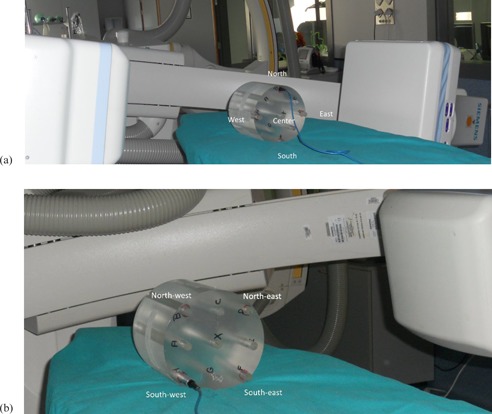
Perspex CT Radcal model 20CT6 head phantom (a) with a central axial hole to measure the central dose, and four axial holes of the phantom at a depth of 1 cm from the surface to measure the peripheral doses (north, west, south, and east). The phantom was rotated 45° (b) to measure another four peripheral doses (northwest, southwest, southeast, and northeast).

In CBCT mode, radiation exposure was assessed with the dose metric (D[0]) proposed by Fahrig et al.^(18)^ D(0) is defined as the average dose over the central phantom plane, using the same area averaging approximation used in conventional CT (${\text{CTDI}}_{w}$). The dosimetry setup is illustrated in Fig. 3. The Perspex CT Radcal model 20CT6 head (Radcal Corp.) and 20CT14 body phantoms (Imperial Chemical Industries, London, U.K.) include five boreholes (labeled north, west, south, east, and center in Fig. 3(a)). The phantom was placed with the measurement positions at north, west, south, east, and center (Fig. 3(a))) and then rotated 45° to measure at northeast, southeast, southwest, and northwest (Fig. 3(b)). The dose measured in the central axial hole of the dosimetry phantom (D0) was weighted by one‐third and added to the average dose measured in eight axial holes of the phantom at a depth of 1 cm from the surface ($D_{p}$), weighted by two‐thirds. These point doses were measured using a PTW UNIDOS electrometer (PTW, Freiburg, Germany) and a PTW TM30001 (0.6 cc) ion chamber calibrated at an accredited laboratory. The ionization chamber energy dependence was ≤2% for all the beam qualities measured. All the dose values are represented in phantom doses in units of air kerma.^18^, ^19^

### E. Dosimetry comparison between cone‐beam CT and cine mode

**Figure 4:**
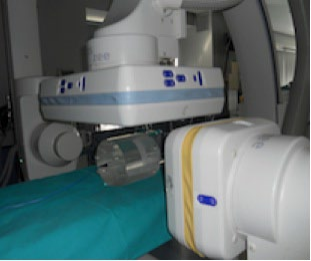
Biplane acquisition setup using CT head phantom.

Several runs were acquired to compare the air kerma‐area product ($P_{\text{ka}}$) and incident air kerma at the patient entrance reference point (Ka,r)^(10)^ measured with the ionization transmission chambers integrated into the collimator housing for the various acquisition modes. The patient entrance reference point is a point along the central ray of the X‐ray beam, 15 cm back from the isocenter toward the focal spot.^(20)^ The 5 s rotational image acquisition run (133 frames) was compared with the cine biplane series, acquiring 133 frames per plane. A biplane series is the simultaneous acquisition of anteroposterior and lateral projections. To measure the series, the head phantom was employed in a clinical practice setup (Fig. 4). For rotational acquisition, the center of the phantom was positioned at the C‐arm isocenter (source‐detector distance, 120 cm; source‐isocenter distance, 75 cm). For biplane acquisition, the table height was kept constant, and the image detectors were moved to a source‐detector distance of 109 cm and 94 cm for lateral and anteroposterior acquisition, respectively, to simulate clinical conditions commonly used at our facility. A 48 cm FOV and a 25 cm FOV were selected for tubes A and B, respectively. $P_{\text{ka}}$ and Ka,r were corrected using the appropriate measured calibration factors to take into account the radiation attenuation by the table and mattress when the frontal C‐arm was used. The $P_{\text{ka}}$ meter was verified *in situ* using a calibrated ionization chamber (Radcal 10×5−60). The calibration procedure and measurement of the beam area were conducted according to the recommendations of the International Atomic Energy Agency protocol.^(21)^ The calibration coefficients varied by ±15%, with an uncertainty of less than 2%.

### F. Cone‐beam CT image quality

The phantom used to evaluate the image quality for CBCT was the 20 cm diameter Catphan 504 (Phantom Laboratories, Salem, New York).^(22)^ The digital images were analyzed by a set of ImageJ macros designed for this purpose.^(23)^ The phantom was divided into several modules containing various test objects. The Catphan CTP404 module has sensitometer targets constructed from Teflon, Delrin, acrylic, polystyrene, polymethylpentene (PMP), low‐density polymethylpentene (LDPE), and air. In this module, Hounsfield units (HU) are measured as the mean pixel value of a circular ROI with a 4 mm radius centered in these materials. The CTP528 high‐resolution module has a 1 through 21 line pair per cm high‐resolution test. At 10 mm from the center, the CTP528 module has a bead point source to measure modulation transfer function (MTF). CTP515 is a low‐contrast module with 2 to 15 mm diameter objects and nominal contrast levels from 0.3% to 1%. CTP486 is the image uniformity module made from solid water to measure spatial uniformity and noise. The integral nonuniformity (UI) is defined over five squared ROIs located in the center and periphery of the image in the four cardinal points as follows:

(3) $$UI=ROI_{\text{max}}-ROI_{\text{min}}$$

where ${\text{ROI}}_{\text{max}}$ and ${\text{ROI}}_{\text{min}}$ are the maximum and minimum ROI mean pixel values. We evaluate another parameter (C) that compares the mean pixel value in the center and periphery of the image. C is defined as follows:

(4) $$C=ROI_{c}-ROI_{p}$$

where ${\text{ROI}}_{c}$ and ${\text{ROI}}_{p}$ are the center and periphery ROI mean pixel values. The standard deviation is measured in a 128×128 pixel ROI placed in the center of the image. A complete description of the CBCT image noise was addressed with the evaluation of the noise power spectrum (NPS), using the images of the uniform section of the Catphan phantom and an in‐house macro programmed in ImageJ,^(15)^ available for free from the authors. NPS provides both the amount and spatial correlation of the noise.

**Figure 5:**
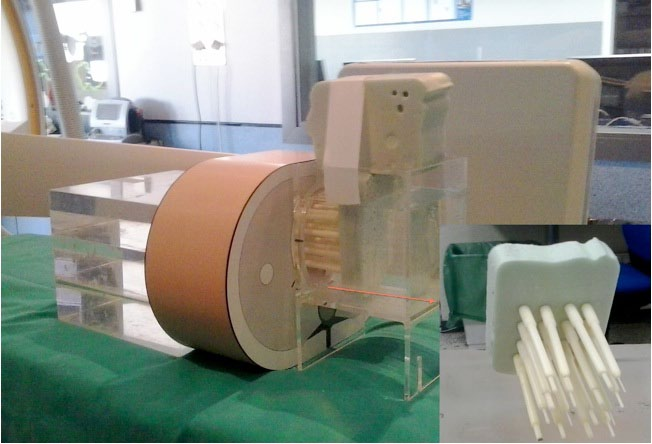
QRM anthropomorphic cardio phantom. The 1 cm rods had diameters that varied from 1 to 4 mm, in 1 mm steps, and contrast levels of 200, 250, 300, and 400 HU.

To assess the visualization of tiny vessels of varying luminal diameters related to contrast medium concentration in CBCT, another anthropomorphic cardio phantom (QRM GMBH, Erlangen, Germany)^(24)^ was imaged. The phantom's dimensions were 30 cm in width, 20 cm in height, and 10 cm in length. The phantom was composed of four parts: a thorax with artificial lung lobes, a spine insert, a soft‐tissue shell of equivalent material, and a cylindrical water tank where rods that simulate vessels can be inserted. The latter are useful for contrast resolution measurements. The rods are 1 cm long and have diameters that vary from 1 to 4 mm in 1 mm steps. The contrast levels of the rods were 200, 250, 300, and 400 HU (Fig. 5).

After image acquisition, projection images were sent to a Siemens Leonardo workstation and reconstructed with syngo DynaCT software (VB15DP01rev1.0 Siemens). The CBCT images were recorded with a 512×512 reconstruction matrix (14 bits) and a 0.46 mm slice width. The employed reconstruction kernels were a normal convolution kernel for 5sDR‐L and a smooth kernel for 5sDRc. For the QRM phantom, the axial images were reconstructed with various slice thicknesses from 0.5 to 8 mm.

## III. RESULTS

### A. Dosimetry and image quality for cine and fluoroscopy modes

Tables 1 to 4 show the most relevant radiographic parameters adjusted by the X‐ray system for fluoroscopy and cine image acquisition modes for the employed protocols and FOVs.

**Figure 6:**
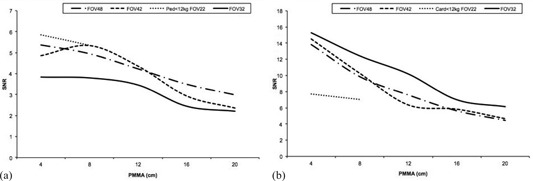
Signal‐to‐noise ratio (SNR) results for (a) the various fluoroscopy examination protocols, and (b) the various cine examination protocols, as well as FOVs evaluated as a function of PMMA thicknesses.

**Figure 7:**
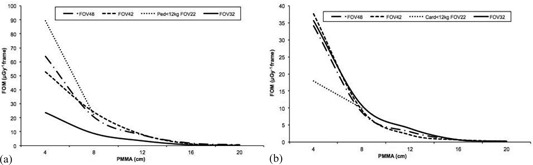
Figure of merit (FOM) ($\mu {\text{Gy}}^{-1}$ frame) results for: (a) the various fluoroscopy examination protocols, and (b) the various cine examination protocols, as well as FOVs evaluated as a function of PMMA thicknesses.

**Figure 8:**
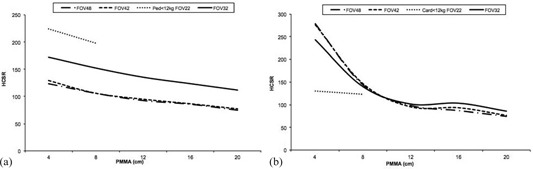
High‐contrast spatial resolution‐related parameter (HCSR) results for: (a) the various fluoroscopy examination protocols, and (b) the X‐ray system for the various cine examination protocols, as well as FOVs evaluated as a function of PMMA thicknesses.

Figures 6, 7, and 8 summarize the numerical values of the image quality parameters measured for fluoroscopy and cine acquisition modes and examination protocols.

Figure 6(a) shows a slight reduction in SNR for thicker phantoms except for FOV 42 cm and PMMA thickness between 4 to 8 cm that the SNR increases. For FOV of 32 cm and PMMA thickness between 4 and 12 cm, SNR is almost constant. The best SNR for the images taken for the various examination protocols is obtained for Ped <12 kg for 4 and 8 cm PMMA. SNR shows a greater variability in cine mode than in fluoroscopy mode (Fig. 6(b)). The highest values for the SNR were obtained for FOV 32 cm for all PMMA thicknesses, whereas the poorest values for 4 and 8 cm PMMA correspond to Card <12 kg protocol and FOV 22 cm.

Figure of merit (FOM) relates the necessary dose to obtain a certain image quality. For phantom thicknesses ranging from 4 to 8 cm of PMMA (using Ped <12 kg and FOV 22 cm fluoroscopy mode), FOM improves over the other fluoroscopy modes measured (Fig. 7(a)). For cine mode this trend is reversed: the poorest FOM is obtained for Card <12 kg and FOV 22 cm for 4 and 8 cm PMMA (Fig 7(b)). FOM decreases with the increment of PMMA thicknesses.

Figure 8(a) shows the tendency for fluoroscopy modes of the high‐contrast spatial resolution parameter to degrade when the thickness of the phantom increases due to increased scatter and when FOV increases. For cine mode, the high‐contrast spatial resolution parameter worsens when the thickness of the phantom increases with a steep descent between 4 and 8 cm of PMMA, but without significant differences between FOV 42, 42, and 32 cm and LV3040 cine mode. For FOV 22 cm and Card <12 kg cine mode and 4 and 8 cm PMMA, HCSR is almost constant.

**Table 1:** Parameters of the examination protocols and acquisition modes employed for a field of view of 48 cm

*Examination Protocol*	*Acquisition Mode*	*Frames per s*	*PMMA*	*Tube Potential (kVp)*	*Tube Current (mA)*	*Copper Filter* ^a^ *(mm)*	*Pulse Width (ms)*	*ESAK (μGy/fr)*
FL3040	FL	3	4	65	16.1	0.6	3.1	0.45
	LV3040	30		63	11	0	3.0	5.62
FL3040	FL	3		65	40.5	0.6	3.3	1.19
	LV3040	30	8	73	14.2	0	3.0	11.03
FL3040	FL	3	12	65	75.7	0.6	3.3	2.36
	LV3040	30		73	28.9	0	3.3	19.16
FL3040	FL	3	16	65	97.7	0.6	7.2	6.85
	LV3040	30		73	85.1	0	3.4	49.28
FL3040	FL	3	20	65	176.6	0.6	8.2	14.06
	LV3040	30		73	177.3	0	3.4	102.5

a ^a^ The filter indicates the mm of Cu automatically added by the system for each acquisition mode and PMMA thickness. ESAK=,entrance surface air kerma; FL,=fluoroscopy mode; LV=cine mode; PMMA=polymethyl methacrylate.

**Table 2:** Parameters of the examination protocols and acquisition modes employed for a field of view of 42 cm and pediatric patients under 12 kg

*Examination Protocol*	*Acquisition Mode*	*Frames per s*	*PMMA*	*Tube Potential (kVp)*	*Tube Current (mA)*	*Copper Filter (mm)*	*Pulse Width (ms)*	*ESAK (μGy/fr)*
FL3040	FL	3	4	65	15.8	0.6	3.1	0.45
	LV3040	30		63	11.1	0	3.0	5.57
FL3040	FL	3	8	65	41.3	0.6	3.3	1.19
	LV3040	30		73	14.4	0	3.1	11.16
FL Ped<12kg	FLPed<12kg	10	4	65	15.8	0.6	3.0	0.46
	Card^a^ <12kg	30		62	73.5	0.6	3.4	1.53
FL Ped<12kg	FL Ped<12kg	10	8	66	34.1	0.6	3.2	1.15
	Card<12kg	30		62	168.9	0.6	3.5	4.02
FL3040	FL	3	12	65	76.9	0.6	3.3	2.4
	LV3040	30		73	29.4	0	3.3	18.97
FL3040	FL	3	16	65	97.8	0.6	7.3	6.75
	LV3040	30		73	89.3	0	3.4	50.33
FL3040	FL	3	20	65	185.4	0.6	8.2	15.26
	LV3040	30		73	186.5	0	3.5	105.67

a ^a^ Card indicates cine mode.

**Table 3:** Parameters of the examination protocols and acquisition modes employed for a field of view of 32 cm and pediatric patients under 12 kg

*Examination Protocol*	*Acquisition Mode*	*Frames per s*	*PMMA*	*Tube Potential (kVp)*	*Tube Current (mA)*	*Copper Filter (mm)*	*Pulse Width (ms)*	*ESAK (μGy/fr)*
FL3040	FL	3	4	65	24.6	0.6	3.2	0.62
	LV3040	30		67	11.2	0	2.9	6.56
FL3040	FL	3	8	65	61.9	0.6	3.3	1.65
	LV3040	30		73	22.9	0	3.3	14.32
FL Ped<12kg	FLPed<12kg	10	4	66	19.7	0.6	3.2	0.6
	Card<12kg	30		62	106	0.6	3.4	2.09
FL Ped<12kg	FL Ped<12kg	10	8	66	49.9	0.6	3.3	1.57
	Card<12kg	30		62	247.6	0.6	3.5	5.55
FL3040	FL	3	12	65	97.6	0.6	4.1	3.46
	LV3040	30		73	46.7	0	3.4	26.07
FL3040	FL	3	16	65	140	0.6	8.2	10.28
	LV3040	30		73	143.2	0	3.4	74.11
FL3040	FL	3	20	66	243.1	0.6	8.2	20.77
	LV3040	30		73	311.2	0	3.5	163.56

**Table 4:** Parameters of the examination protocols and acquisition modes employed for a field of view of 22 cm and pediatric patients under 12 kg

*Examination Protocol*	*Acquisition Mode*	*Frames per s*	*PMMA*	*Tube Potential (kVp)*	*Tube Current (mA)*	*Copper Filter (mm)*	*Pulse Width (ms)*	*ESAK (μGy/fr)*
FL3040	FL	3	4	65	36.3	0.6	3.3	0.81
	LV3040	30		73	12.3	0	3.0	8.47
FL3040	FL	3	8	65	93.1	0.6	3.3	2.26
	LV3040	30		73	35.7	0	3.3	18.32
FL Ped <12kg	FLPed<12kg	10	4	66	29.8	0.6	3.2	0.79
	Card<12kg	30		62	155.4	0.6	3.4	2.85
FL Ped <12kg	FL Ped<12kg	10	8	66	76.4	0.6	3.3	2.16
	Card<12kg	30		62	397.8	0.6	3.5	8.16

### B. Cone‐beam CT mode dosimetry

Table 5 shows the dose measurement results for the 16 cm and 32 cm diameter dose phantoms for the two analyzed 3D acquisition protocols: a standard (5sDRc) and a low‐dose mode (5sDR‐L). Numerical values represent the mean and standard deviation of three measurements at each location.

**Table 5:** Measurements for the 16 cm and 32 cm diameter dose phantoms and cone‐beam CT protocols

*Phantom*	*Protocol*	${\text{kV}}_{p}$	*Total mAs*	$D_{o}$ *(mGy)*	$D_{p}$ *(mGy)*	*D(0) (mGy)*	$P_{\text{ka}}$ *(μGy.m^2^)*	$K_{a,r}$ *(mGy)*
Head	5sDR‐L	67	75.9±0.0	1.01±0.00	1.76±0.78	1.52±0.51	104.3±0.1	4.6±0.1
	5sDRc	90	50.9±0.2	2.78±0.00	5.04±2.19	4.29±1.46	305.1±0.1	13.7±0.1
Body	5sDR‐L	87	402.2±0.0	5.66±0.01	14.28±9.57	11.40±6.38	1036.6±0.7	46.6±0.1
	5sDRc	95	789.4±0.8	19.23±0.05	54.86±36.59	42.98±24.40	4539.4±17.9	204.0±1.1

### C. Dosimetry comparison between cone‐beam CT and cine mode

Table 6 shows the $P_{\text{ka}}$ and Ka,r values measured by the system, corrected by the appropriate calibration factor, for 133 frame acquisition with various protocols and head (16 cm diameter) phantom for planes A and B. The rotational acquisition was performed with the frontal C‐arm (plane A). FOVs of 48 cm and 25 cm without collimation were selected for tubes A and B, respectively.

**Table 6:** $P_{\text{ka}}$ and Ka,r and rotational modes. measured by the system if 133 images of the head phantom were acquired in the various cine

*Mode*	*Protocol*	*Plane*	$P_{\text{ka}}$ *(μGy.m^2^)*	Ka,r *(mGy)*
Rotational	5sDR‐L	A	104.3	4.6
	5sDRc	A	305.1	13.7
	Card<12kg	A	65.1	1.7
B		28.9	4.2
A+B		94.0	5.9
Cine	LV3040	A	170.5	4.8
		B	60.3	8.6
		A+B	230.8	13.4

### D. Cone‐beam CT image quality

Table 7 shows the HU of each material in the CTP404 module of the Catphan 504 phantom measured in the CBCT images, acquired with standard‐dose (5sDRc) and low‐dose (5sDR‐L) protocols (see Fig. 10(a)).

**Figure 9:**
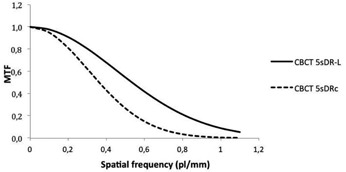
MTF function of the cone‐beam CT Catphan bead images obtained with protocols 5sDRc and 5sDR‐L and field of view 48 cm.

**Figure 10:**
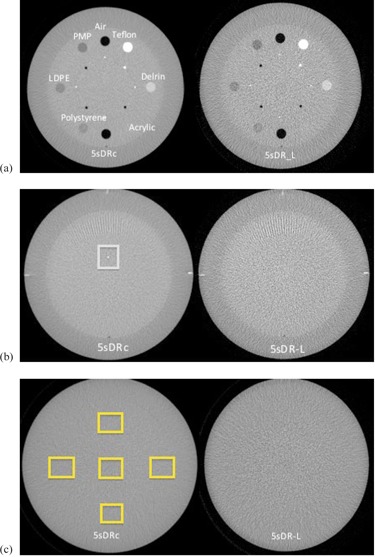
Catphan phantom cone‐beam CT images of various modules: (a) CTP404 module with various material targets; (b) bead point source on CTP528 module; (c) CTP515 image uniformity module. The left image was acquired with the 5sDRc standard‐dose protocol, and the right image was acquired with the low‐dose protocol 5sDR‐L.

Figure 9 shows the MTF obtained in the CBCT Catphan bead images for a FOV of 48 cm (see Fig. 10(b)).

The low‐contrast sensitivity module could not be analyzed because the low‐contrast inserts from the Catphan CTP515 module were indistinguishable from its background in the CBCT 0.46 mm slice width images.

Table 8 shows the UI and C parameters and the standard deviation measured in a 128×128 pixel ROI in the image center of the uniform module of the Catphan phantom for the two analyzed CBCT protocols (see Fig. 10(c)).

**Table 7:** Hounsfield units (HU) of each material in the CTP404 module of the Catphan 504 phantom measured in cone‐beam CT images

	*Protocol*	*5sDRc*	*5sDR‐L*
*Material*	*Reference HU*	*(HU)*	
Air	−1016	−880	−864
PMP^a^	−196	−204	−219
LDPE^b^	−104	−137	−146
Polystyrene	−47	−93	−104
Acrylic	114	41	32
Delrin	365	245	252
Teflon	1000	799	854

a ^a^ PMP polymethylpentene.

b ^b^ LDPE low‐density polymethylpentene.

**Figure 11:**
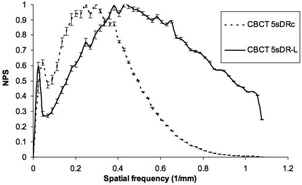
Normalized noise power spectrum for the cone‐beam CT (48 cm FOV) images. Each spectrum is normalized to its maximum.

We computed the 2D‐NPS using the CBCT axial images for 5sDRc and 5sDR‐L protocols and for FOV 48 cm (the results for smaller FOV are equivalent). We only show a one‐dimensional NPS obtained as the average of the NPS radial profiles because the acquired NPS presented rotational symmetry. Figure 11 shows the normalized NPS for the CBCT images. Each NPS was normalized to its maximum to make a direct comparison of the noise structures, despite the large differences in their size.

**Figure 12:**
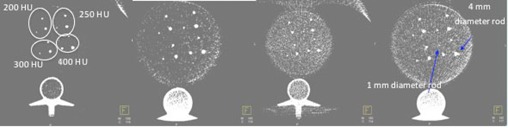
Axial QRM Phantom images. The first and second images on the left were acquired with the CBCT 5sDRc protocol and 48 and 42 cm FOVs. Both images were reconstructed with a slice width of 0.5 mm. In both images, we can distinguish 2 mm diameter rods with 200, 250, and 300 HU and a 1 mm diameter rod with 400 HU. The last two images were acquired with the CBCT 5sDR‐L protocol and 48 and 42 cm FOVs. The images were reconstructed with 0.5 and 6 mm slice widths. We can distinguish a 2 mm diameter rod with 400 and 300 HU and a 3 mm diameter rod with 250 and 200 HU in the first image. In the last image, we can see a 1 mm diameter rod with 400 and 300 HU and a 2 mm diameter rod with 250 and 200 HU.

Table 9 and Figure 12 show the visibility of the rods with various diameters and contrast levels in axial images of the QRM phantom. The acquisition parameters $P_{\text{ka}}$ and Ka,r are also shown. The images were acquired with the two CBCT protocols and the two allowed FOVs. The images were reconstructed with slice widths between 0.5 and 8 mm. Window level and width was approximately 118 and 139 for all the evaluated images.

**Table 8:** Uniformity index (UI), C parameter, and standard deviation (SD) measured in a 128×128 pixel ROI measured in the uniform section of the Catphan phantom

*Examination Protocol*	*UI (HU)*	*C (HU)*	*SD*
5sDRc	21.31	−6.21	36.1
5sDR‐L	12.18	−4.74	160.9

**Table 9:** QRM phantom rod visibility

*Protocol*	*FOV (cm)*	*kVp*	*Total mAs*	$P_{\text{ka}}$ *(μGy.m^2^)*	Ka,r *(mGy)*	*Rod Visibility*
5sDRc	48	90	51.37±0.10	310.9±1.2	14.4±0.1	1 mm diameter rod and 400 HU
						2 mm diameter rod and 300,250 and 200 HU
						… for 0.5, 2, 6, and 8 mm slice width
5sDRc	42	90	52.95±0.13	248.2±1.1	14.9±0.1	1 mm diameter rod and 400 HU
						2 mm diameter rod and 200, 250, and 300 HU
						. for 0.5, 2, 6, and 8 mm slice width
						2 mm diameter rod and 300 and 400 HU
						3 mm diameter rod and 250 and 200 HU
						. for 0.5 mm slice width
5sDR‐L	48	67	79.02±0.22	108.9±0.7	5.0±0.0	2 mm diameter rod and 200, 250, 300 and 400 HU
						. for 2 mm slice width
						2 mm diameter rod and 200, 250 and 300 HU
						1 mm diameter rod and 400 HU
						. for 6 and 8 mm slice width
						2 mm diameter rod and 250, 300, and 400 HU
						3 mm diameter rod and 200 HU
						. for 0.5 mm slice width
5sDR‐L	42	67	80.65±0.03	86.4±0.3	5.2±0.0	2 mm diameter rod and 200, 250, 300 and 400 HU
						. for 2‐mm slice width
						2 mm diameter rod and 200 and 250 HU
						1 mm diameter rod and 300 and 400 HU
						. for 6 and 8 mm slice width

## IV. DISCUSSION

### A. Cine and fluoroscopy mode dosimetry and image quality

In order to properly characterize X‐ray imaging systems, basic information on the dose per frame for the various modes of operation (fluoroscopy and cine) must be obtained. Another important piece of information is the dependence of the dose per frame on the phantom thickness (patient size) for the various modes of operation. The dose values need to be balanced with the image quality for the various operation modes. Thus, a visual assessment of the images is insufficient to determine whether the default settings of the X‐ray systems are optimal for the various patient sizes or whether changes can be performed to obtain a similar image quality with lower patient doses. Numerical assessments using test object images can help optimize these settings. These measurements and other values reported in the literature^4^, ^8^, ^25^, ^26^ should be communicated to cardiologists to help optimize clinical protocols.

As expected, the radiographic parameters (kVp and mA) adjusted by automatic exposure control increased with PMMA thicknesses (Tables 1 to 4). The ESAK values for the fluoroscopy mode ranged from 0.6 to 20.8 μGy/frame when the PMMA thickness was increased from 4 to 20 cm for FOV 32 cm. For the cine mode, the ESAK values ranged from 6.6 to 163.6 μGy/frame for the same FOV and LV3040 mode. An increase in patient thickness from 4 to 8 cm resulted in a 2.7‐fold increase in the entrance phantom dose in the fluoroscopy mode. An increase from 8 to 12 cm resulted in a twofold increase, an increase from 12 to 16 cm resulted in a threefold increase, and an increase from 16 to 20 cm resulted in a twofold increase. In cine mode, the corresponding dose increases were 2, 1.7, 2.6, and 2. An important aspect for cardiologists to consider is the three to twelvefold increase in the ESAK/frame when comparing cine with fluoroscopy frames. This system can store fluoroscopy runs in DICOM format; therefore, if high image quality is not required, a fluoroscopy run should be considered as an option for documenting part of the procedure.^(4)^ For 22 cm FOV and 4 and 8 cm PMMA, the selection of Ped <12 kg instead of FL3040 will reduce the ESAK/frame for the cine mode threefold and 2.2‐fold, respectively, for the same fluoroscopy ESAK/frame.

Image quality was evaluated using three numerical parameters: SNR, FOM, and HCSR decrease as phantom thickness increases. If the PMMA thickness increases from 4 to 8 cm, the system raises the tube voltage, yet SNR (Fig. 6) drops because of decreasing primary contrast, detector efficiency, and increasing scatter fraction. For PMMA thicknesses above 8 cm, the generator is programmed to increase mA first to preserve contrast, but at the cost of increased dose to the patient. This is due to the logic applied by the X‐ray system for the various protocols evaluated for procedures where contrast is critical. Tube voltage, copper filter, and pulse width variations are limited depending on the protocol selection to avoid image quality degradation. The FOM parameter has been employed by other authors^16^, ^27^ to evaluate the cost (in terms of dose per frame) of obtaining a given image quality. The FOM shows a tendency to decrease with increasing PMMA thickness (Fig. 7), meaning that the image quality decreases significantly when the phantom thickness increases, and the FOM employed is only useful for a certain range of image quality and dose per frame. Figure 8 shows that HCSR decreases smoothly as phantom thickness increases due to the influence of scatter radiation.

The variation in the FOV parameters is related to how the system acquires the signal. For the fluoroscopy mode and 48 and 42 cm FOVs, the system uses a binned mode, whereas magnification for 32 and 22 cm FOV uses unbinned pixels. The binning principle implies combining 2 pixels in the horizontal and 2 pixels in the vertical direction into one on the flat detector to be read as single line of data, thereby obtaining a higher SNR but lower spatial resolution. In cine mode, the binned mode is employed for 48, 42, and 32 cm FOVs. Table 10 shows the matrix pixel size and pixels per mm for the various FOVs and acquisition modes. The largest difference between SNR in fluoroscopy and cine occurs for 32 cm FOV because fluoroscopy is acquired with a 1440×1440 pixel matrix size in unbinned mode, whereas cine is acquired at 720×720 pixels in binned mode. As a result, the fluoroscopy mode with a 32 cm FOV yields the lowest SNR. The SNR in fluoroscopy mode is lower than that for cine mode. For 4 and 8 cm PMMA, the highest SNR for fluoroscopy is obtained if FL Ped <12 kg is selected.

The FOM diminishes when the FOV is changed from 48 to 32 cm in the fluoroscopy mode. Fluoroscopy mode with 32 cm FOV yields the lowest SNR and to the lowest FOM because magnification in fluoroscopy for the 32 cm FOV uses unbinned pixels. The highest FOM for fluoroscopy is achieved for 22 cm FOV and 4 cm PMMA in the Ped <12 kg protocol. In the cine mode, the FOM remains almost constant as the FOV changes from 48 to 32 cm. The FOM should only be used to compare images taken in the same acquisition mode (cine or fluoroscopy) due to the large differences in noise in these acquisition modes.^(28)^ In fluoroscopy mode, HCSR increases as the FOV decreases. This is because magnification in fluoroscopy is performed with unbinned pixels. In cine mode, magnification is performed electronically from 48 to 32 cm FOV, therefore no increase in resolution is observed. The line pair resolution for a 22 cm FOV and pediatric protocols is higher for fluoroscopy mode than for cine mode. This is because tube voltage for 4 and 8 cm PMMA are similar for the two modes; however, in the fluoroscopy mode, a 0.6 mm focal spot size is selected instead of the 1 mm focal spot size for use with cine mode.

**Table 10:** Image sizes and modes (binned, unbinned) for various acquisition modes and fields of view

*Acquisition Mode*	*FOV (cm)*	*Image Size* width×height, *pixels (mode)*	*Pixels/mm*
Fluoroscopy Cine	48	1240×960 (binned mode)	3.247
Fluoroscopy Cine	42	960×960 (binned mode)	3.247
Fluoroscopy	32	1440×1440 (unbinned mode)	6.494
Cine		720×720 (binned mode)	3.247
Fluoroscopy Cine	22	1024×1024 (unbinned mode)	6.494

### B. Dosimetry comparison between cone‐beam CT and cine mode

Acquiring CBCT series with a 16 cm head phantom and a standard‐dose protocol results in a threefold increase in D(0), $P_{\text{ka}}$ and $P_{a,r}$, compared with the low‐dose protocol. This increase is higher in the 32 cm body phantom (Table 5).

Biplane acquisition of 133 frames with the head phantom in Card <12 kg cine mode implies a similar $P_{\text{ka}}$ compared with a 3D run in the low‐dose protocol (5sDR‐L), but with a higher $P_{a,r}$. When the LV3040 protocol was selected during head phantom biplane acquisition, a 24% reduction in $P_{\text{ka}}$ was achieved compared with 5sDRc CBCT with similar $P_{a,r}$ (Table 6). The difference with CBCT is that the skin dose is distributed over the various projection angles. Sometimes a biplane angiography is insufficient to obtain relevant anatomic information. A CBCT can aid in complex catheter manipulation where the 3D angiography image is used as an overlay on a fluoroscopy screen; so if both runs are taken, radiation doses should be considered. Sometimes a standard angiography is needed after a CBCT due to limitations in the temporal resolution of CBCT.

### C. Cone‐beam CT image quality

Table 7 shows that the measured HU values do not agree with the reference values for CBCT images. These inaccuracies are due to the increased scatter generated by the CBCT. A greater quantity of scattered X‐rays than conventional CT is produced in CBCT, thus its ability to detect low‐contrast tissue is reduced due to enhanced noise in reconstructed images. Another limitation of CBCT is beam hardening, which influences the density values. Cardiologists can select the center and width of the visualization window to better visualize tissues. The absolute value of the HU is not relevant because the cardiologists work subjectively.

CBCT images acquired with 5sDR‐L and a normal filter present better spatial resolution at all frequencies than those acquired with the 5sDRc protocol and a smooth filter due to the smaller focal spot used with the 5sDR‐L protocol (Fig. 9). The low‐dose protocol uses less kVp and a small focal spot size (0.6 mm), compared with the 1 mm focal spot size and approximately 90kVp for the standard‐dose protocol.

The CBCT images reveal the presence of a cupping effect; the central part of the image is hypodense compared with the periphery. The effect can be explained by inaccurate beam hardening corrections (Table 8 and Fig. 10(c)).

The amount of noise present in the low‐dose CBCT images is much higher than that obtained with the 5sDRc mode (Table 8 and Fig. 10). The standard deviation in a 128×128 pixel ROI in the image center of the uniform module of the Catphan phantom is approximately 4.5‐fold higher in the image acquired with the low‐dose protocol. Collimation from top to bottom (before performing the 3D rotation) is allowed but is not typically used because, in some cases, extra‐cardiac vascular structures must be imaged. However, collimation should be applied whenever possible to reduce unnecessary radiation and image noise.

In Fig. 11, we can see that the 5sDR‐L NPS presents an important shift of the spectrum to higher frequencies and does not show the high frequency roll‐off. This shift is consistent with the image reconstruction filters that the manufacturer applies to the 5sDRc (smooth) and 5sDR‐L (standard) modes. The nonzero value of the 5sDR‐L NPS (and MTF) at the cutoff frequency indicates the presence of aliasing.^(29)^ Finally, the low frequency peaks present in the teo NPS shown are due to structural noise.

Figure 12 and Table 9 show that we are capable of distinguishing a 1 mm diameter rod with 250 HU if we reconstruct 5sDR‐L 48 cm FOV images with an 8 mm slice width. An optimal attenuation for coronary angiography is 250–300 HU.^(30)^ High‐contrast structures can therefore be imaged during arteriographic procedures.

The entire analysis for the fluoroscopy, cine, and CBCT quality images was performed in a static mode (patient movement was not taken into account), which could be considered a limitation. Caution is required whenever a device is to be used, given that rotational images offer an average measurement of cardiac and vessel diameters, and there is wide variation between systole and diastole throughout the cardiac cycle.

## V. CONCLUSIONS

We have performed a dose and image quality characterization of the biplane system with 3D rotational angiography capability. This study has shown that proper characterization of the equipment requires full awareness of its technical features and operating modes, especially those related to image quality, given that measurements can provide unexpected results. The results presented are intended for the implementation of dose‐reduction techniques with minimal loss of image quality.

The system's ability to store fluoroscopy runs enables radiologists to replace cine with the fluoroscopy mode if sufficient image quality is achieved. This mode could be used to document procedures, resulting in significant dose savings. To find a balance between noisy and HCSR images, it is important to know how the system works as the FOV changes.

Here are a number of practical guidelines. When examining children with a chest thickness of less than 12 cm, a fluoroscopy protocol of Ped <12 kg should be selected. These runs should be stored instead of acquiring cine runs with a Card<12 kg protocol. This enables the acquisition of sufficient (or better) quality images with a lower dose. If cine acquisition is required, the cardiologist should know that the highest SNR is obtained with the LV3040 protocol and 32 cm FOV. Although the FL3040 protocol yields the lowest SNR with fluoroscopy, it provides the highest HCSR for patients with a chest thickness greater than 12 cm.

In conclusion, the system offers a novel 3D imaging mode. The acquisition of CBCT images results in increased doses administered to the patients, but also provides further diagnostic information contained in the volumetric images. The assessed CBCT protocols provide images that are noisy but with very good spatial resolution. High‐contrast structures can, therefore, be imaged during arteriographic procedures.

Other effective radiation dose reduction techniques not assessed in this study, such as collimation and antiscatter grid remove for neonates, should be implemented.

## ACKNOWLEDGMENTS

The authors would like to thank the Spanish Nuclear Safety Council for their support in the framework of the 2012–2014 call for projects on Radiological Protection. The authors would also like to thank Ruben Larbec and Ramon Garcia from Siemens Healthcare Madrid for their technical assistance.

## COPYRIGHT

This work is licensed under a Creative Commons Attribution 3.0 Unported License.

## References

[1] International Commission on Radiological Protection (ICRP) . Avoidance of radiation injuries from medical interventional procedures. ICRP Publication 85. Ann ICRP. 2000;30(2):7–67. 10.1016/S0146-6453(01)00004-5 11459599

[2] European Community (EC) . Council Directive of 5 December 2013 (2013/59/Euratom) laying down basic safety standards for protection against the dangers arising from exposure to ionising radiation. Luxemburg: EC; 2014. 10.7417/CT.2024.5127 39400087

[3] European Community (EC) . Criteria for acceptability of medical radiological equipment used in diagnostic radiology, nuclear medicine and radiotherapy. Radiation Protection No. 162. Luxemburg: European Commission. Directorate General for Energy, Nuclear Safety and Fuel Cycle; 2012.

[4] Vano E , Ubeda C , Leyton F , Miranda P . Radiation dose and image quality for paediatric interventional cardiology. Phys Med Biol. 2008;53(15):4049–62. 18612174 10.1088/0031-9155/53/15/003

[5] Cousins C , Miller DL , Bernardi G et al. ICRP Publication 120: Radiological protection in cardiology. Ann ICRP. 2013;42(1):1–125. 10.1016/j.icrp.2012.09.001 23141687

[6] Jones AK , Balter S , Rauch P , Wagner LK . Medical imaging using ionizing radiation: optimization of dose and image quality in fluoroscopy. Med Phys. 2014;41(1):014301. 24387534 10.1118/1.4835495

[7] Corredoira E , Vañó E , Ubeda C , Gutierrez‐Larraya F . Patient doses in paediatric interventional cardiology: impact of 3D rotational angiography. J Radiol Prot. 2015;35(1):179–95. 25632824 10.1088/0952-4746/35/1/179

[8] Vano E , Ubeda C , Geiger B , Martinez LC , Balter S . Influence of image metrics when assessing image quality from a test object in cardiac X‐ray systems. J Digit Imaging. 2011;24(2):331–38. 20127268 10.1007/s10278-009-9268-7 PMC3056969

[9] Faulkner K , Malone J , Vano E et al. The SENTINEL project. Radiat Prot Dosimetry. 2008;129(1‐3):3–5. 18310611 10.1093/rpd/ncn019

[10] International Commission on Radiological Units and Measurements . Patient dosimetry for x rays used in medical imaging. ICRU Report 74. J ICRU. 2005;5(2):1–113.

[11] Seissl J , Eschenbacher H . X‐ray diagnostic apparatus with a filter device [patent]. Patent No: US5680435 A. 1997 Available from: http://www.google.com.ar/patents/US5680435

[12] Rassow J , Schmaltz AA , Hentrich F , Streffer C . Effective doses to patients from paediatric cardiac catheterization. Br J Radiol. 2000;73(866):172–83. 10884731 10.1259/bjr.73.866.10884731

[13] TOR 18FG fluoroschopy phantom specification sheet. Boroughbridge, UK: Leeds Test Objects; [n.d.] Available from: http://www.leedstestobjects.com/wp‐content/uploads/TOR‐18FG‐product‐specifications1.pdf

[14] Rasband WS . Image J. Bethesda, MD: US National Institutes of Health; 2006 Accessed 9 December 2009. Available from: http://imagej.nih.gov/ij/download/

[15] Abràmoff MD , Magalhães PJ , Ram SJ . Image processing with Image J. Biophotonics International. 2004;11(7):36–41. Available from: http://dspace.library.uu.nl/bitstream/handle/1874/204900/ImageJ.pdf?sequence=1

[16] Gagne RM , Boswell JS , Myers KJ . Signal detectability in digital radiography: spatial domain figures of merit. Med Phys. 2003;30(8):2180–93. 12945984 10.1118/1.1578485

[17] Massoumzadeh P , Rudin S , Bednarek DR . Filter material selection for region of interest radiologic imaging. Med Phys. 1998;25(2):161–71. 9507475 10.1118/1.598191

[18] Fahrig R , Dixon R , Payne T , Morin RL , Ganguly A , Strobel N . Dose and image quality for a cone‐beam C‐arm CT system. Med Phys. 2006;33(12):4541–50. 17278805 10.1118/1.2370508

[19] Dixon RL , Anderson JA , Bakalyar DM , et al. Comprehensive methodology for the evaluation of radiation dose in x‐ray computed tomography. AAPM Report No 111. Report of Task Group 111: The future of CT dosimetry. College Park, MD: AAPM; 2010 Accessed 27 December 2014. Available from: http://www.aapm.org/pubs/reports/RPT_111.pdf

[20] International Electrotechnical Commission . International Standard ‐ Medical electrical equipment — Part 2‐43: Particular requirements for the safety of X‐ray equipment for interventional procedures. IEC 60601‐2‐43. Geneva: IEC; 2000.

[21] International Atomic Energy Agency (IAEA) . Dosimetry in diagnostic radiology: an international code of practice. Technical Reports Series No. 457. Vienna: IAEA; 2007 Accessed 15 December 2014. Available from: http://www‐pub.iaea.org/MTCD/publications/PDF/TRS457_web.pdf

[22] Catphan 504 manual. Salem, NY: The Phantom Laboratory; 2013 Available from: http://static1.squarespace.com/static/5367b059e4b05a1adcd295c2/t/551ae42be4b046662454b34d/1427825707349/catphan504manual.pdf

[23] Garayoa J and Castro P . A study on image quality provided by a kilovoltage cone‐beam computed tomography. J Appl Clin Med Phys. 2013;14(1):239–57. 10.1120/jacmp.v14i1.3888 PMC5714052 23318380

[24] QRM Thorax Phantom specifications. Moehrendorf, Germany: QRM; [n.d.] Available from: http://www.qrm.de/content/pdf/QRM‐Thorax.pdf

[25] Faulkner K . The DIMOND project and its impact on radiation protection. Radiat Prot Dosimetry. 2005;117 (1‐3):3–6. 16461511 10.1093/rpd/nci700

[26] Nickoloff EL , Strauss KJ , Austin BT et al. Cardiac catheterization equipment performance. AAPM Report No 70. Report of Task Group 17. College Park, MD: AAPM; 2001 Accessed 15 December 2014. Available from: http://www.aapm.org/pubs/reports/RPT_70.pdf

[27] Samei E , Dobbins JT 3rd , Lo JY , Tornai MP . A framework for optimising the radiographic technique in digital X‐ray imaging. Radiat Prot Dosim. 2005;114 (1‐3):220–29. 10.1093/rpd/nch562 15933112

[28] Vano E , Geiger B , Schreiner A , Back C , Beissel J . Dynamic flat panel detector versus image intensifier in cardiac imaging: dose and image quality. Phys Med Biol. 2005;50(23):5731–42. 16306664 10.1088/0031-9155/50/23/022

[29] Kijewski MF and Judy PF . The noise power spectrum of CT images. Phys Med Biol. 1987;32(5):565–75. 3588670 10.1088/0031-9155/32/5/003

[30] Becker CR , Hong C , Knez A et al. Optimal contrast application for cardiac 4‐detector‐row computed tomography. Invest Radiol. 2003;38(11):690–94. 14566178 10.1097/01.rli.0000084886.44676.e4

